# A crop type dataset for consistent land cover classification in Central Asia

**DOI:** 10.1038/s41597-020-00591-2

**Published:** 2020-07-28

**Authors:** Ruben Remelgado, Sherzod Zaitov, Shavkat Kenjabaev, Galina Stulina, Murod Sultanov, Mirzakhayot Ibrakhimov, Mustakim Akhmedov, Victor Dukhovny, Christopher Conrad

**Affiliations:** 1 Institute of Geography and Geology, Julius Maximilian University Wuerzburg, Wuerzburg, Germany; 2 German Central for Integrative Biodiversity Research (iDiv), Leipzig, Germany; 3 Scientific-Information Centre of the Interstate Coordination Water Commission of the Central Asia (SIC ICWC), Tashkent, Uzbekistan; 4 Urgench State University (UrSU), Khorezm Rural Advisory Support Service (KRASS), 14, Khamid Olimjan Street, 220100 Urgench, Khorezm Uzbekistan; 5 United Nations Development Programme (UNDP), Dushanbe Country Office, Dushanbe, Tajikistan; 6 Institute of Geography, Martin Luther University Halle-Wittenberg, Halle, Germany

**Keywords:** Geography, Hydrology, Agriculture, Climate-change mitigation

## Abstract

Land cover is a key variable in the context of climate change. In particular, crop type information is essential to understand the spatial distribution of water usage and anticipate the risk of water scarcity and the consequent danger of food insecurity. This applies to arid regions such as the Aral Sea Basin (ASB), Central Asia, where agriculture relies heavily on irrigation. Here, remote sensing is valuable to map crop types, but its quality depends on consistent ground-truth data. Yet, in the ASB, such data are missing. Addressing this issue, we collected thousands of polygons on crop types, 97.7% of which in Uzbekistan and the remaining in Tajikistan. We collected 8,196 samples between 2015 and 2018, 213 in 2011 and 26 in 2008. Our data compile samples for 40 crop types and is dominated by “cotton” (40%) and “wheat”, (25%). These data were meticulously validated using expert knowledge and remote sensing data and relied on transferable, open-source workflows that will assure the consistency of future sampling campaigns.

## Abstract (summary)

**Table:** 

Measurement(s)	crop type • land cover • Uncertainty • Normalized Difference Vegetation Index
Technology Type(s)	GPS navigation system • satellite imaging • computational modeling technique
Factor Type(s)	year of data collection • crop type label
Sample Characteristic - Environment	agricultural field
Sample Characteristic - Location	Uzbekistan • Tajikistan

Machine-accessible metadata file describing the reported data: 10.6084/m9.figshare.12613775

## Background & Summary

Land cover change is a key driver of climate change^1,2^. It contributes to regional^3^ and global^4^ warming which creates the risk for severe consequences for, among others, water^5,6^ and food^7,8^ security. Therefore, land cover is an essential decision support variable^9^ that supports decision makers, helping develop local, national, and international management policies^10,11^.

Land cover information is particularly important in arid regions such as the Aral Sea Basin (ASB) in Central Asia. Here, agriculture depends heavily on irrigation^12,13^, a crucial element in answering the dietary needs of a growing population^14^. Simultaneously, food security is threatened by water scarcity, a phenomenon created by the over-exploration of water resources and climate change^15–17^. This transforms the region into a geo-political hotspot where international, water-driven conflicts are possible^18,19^ unless mitigated by sustainable water management^20^. Therefore, the stability of the Central Asia region demands the development of sustainable agricultural practices. To achieve this, accurate information on the spatial and temporal distribution of land cover is required, especially that on crop types. Since different crops types have unique irrigation requirements^21^, knowing their temporal and spatial distribution helps optimize water usage and avoid land degradation^22,23^.

**Fig. 1:**
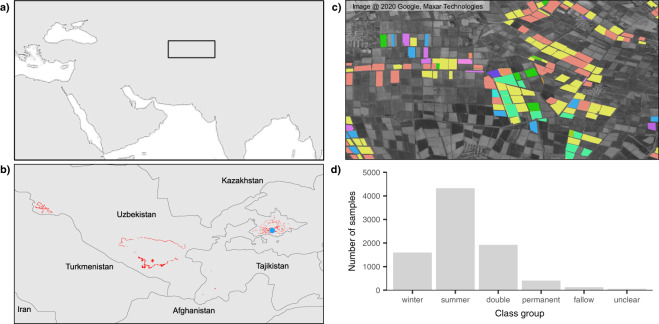
Spatial and phenological distribution of samples. Location of the study region (**a**) described by maximum extent of the collected ground-truth data (**b**). Panel (c) is highlighted in blue in panel (b), and exemplifies the spatial detail of these data within the Fergana, Uzbekistan, collected on a field-by-field basis. These samples depict different crop types which compose distinct phenological class groups (**d**). “Winter” crops are solely represented by winter-wheat while “summer” combines “cotton”, “rice” and “maize” and “permanent” combines “orchards, “vineyards” and “alfalfa”. “Fallow” is described by samples from unused agricultural land, lacking a clear phenological peak. Crop types that lacked a characteristic phenological behaviour (e.g. potatoes, onions) were labelled as “unclear”. “Double” corresponds to “winter” crops that were followed by a “summer” crop after the first harvest.

To map the distribution of crops, remote sensing is a popular and powerful tool. It allows us to map agricultural land cover promptly and efficiently, supporting several global, agricultural monitoring application^24,25^. Still, while remote sensing technologies are powerful, they depend on reliable ground-truth data. When dealing with applications such as land cover mapping, ground-truth data teach us how to distinguish classes and helps us validate their accuracy. However, access to reliable ground information on crops in data scarce regions such as Central Asia is limited. Here, existing field data on crop types is reduced to national surveys that are only available in aggregated tables of questionable quality^26^. In response to this data gap, we compiled a database of 8,435 validated samples on crop types collected within several irrigated cropland areas (Fig. 1a). Of these samples, 97.7% were collected in Uzbekistan and the remaining in Tajikistan. We collected 26 samples in 2008, 213 in 2011 and 8,196 between 2015 and 2018. These data cover 40 classes, 65% of which correspond to “cotton” and “wheat”, reflecting their dominance in the region^27^. “Cotton” is the biggest contributor to this proportion with 40% of the total (Fig. 1d).

We collected these ground-truth data in the scope of the project Central Asia Waters (CAWa, CAWa, www.cawa-project.net) in an effort to provide consistent, timely land cover information on crop types for efficient water management in Central Asia. These data are an input required by the Water Use Efficiency Monitor in Central Asia (WUEMoCA) web service (http://wuemoca.net/app/) and will help improve data services in the region. Consequently, our sample dataset will increase in volume as soon as new field campaigns take place.

## Methods

### Sample collection

**Fig. 2:**
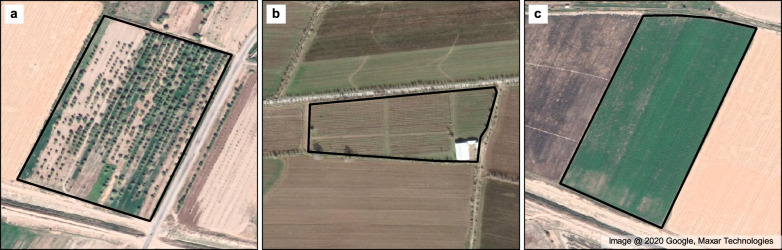
Comparison of field samples. Image (**a**) shows a typical example of a communitarian urban garden. The land parcel is divided between several individuals and used to plant multiple types of crops, including fruit trees. Such cases are hard to interpret with remote sensing due to mixed pixel effects. Image (**b**) depicts a field where crops share the managed areas with buildings. Excluding the building is essential to accurately depict the spectral signature of the cultivated area. Image (**c**) shows an ideal case, where the field was cultivated with a single crop with no additional structures within it. Due to their homogeneity, such fields help discriminate crop specific spectral signatures.

Initially, our crop sample database was composed by points collected with Geographic Positioning Systems (GPS). Most were retrieved close to roads, expressing the poor accessibility within between fields. We collected a single GPS point for each field when either its centre or edges were accessible. When the access to a field was completely obstructed, we collected several GPS points along its borders to aid in its later distinction. After the field survey, we drew polygons around the respective fields through image interpretation. We relied on multi-temporal, very high-resolution satellite imagery from Google Earth (GE). While drawing the extent of each sample, we circumvented non-vegetated areas, excluded samples from fields with only mixed-crops (e.g. urban gardens). Moreover, we excluded samples with no valid GE Data, either due to the lack of observations or due to heavy cloud-cover. The first two criteria address the issue of mixed pixels. While GE shows us a clear demarcation between e.g. a crop and a building within a field, this is often unclear when using satellite data. Satellite sensors commonly used for land cover classification, such as Landsat and the Moderate Resolution Spectroradiometer (MODIS), miss such details due to their coarse spatial resolution. Therefore, when the area within a pixel is composed by different land cover types (i.e. mixed-pixels, Fig. 2a,b), we obtain an unclear spectral signature that will negatively influence the training and validation of a land cover classification. The third criteria avoids uncertainties in our dataset related to land cover change. When drawing polygons, we looked for images within the same year and as close as possible to the sampling date. When no image was available, we considered images within 1 year of the growing season, collected both before and after, given no visible change of the field borders. The polygons vary greatly in size, with areas ranging between 5 m^2^ and 484,507 m^2^. Roughly 75% of samples have an area below 100,000 m^2^ (10 ha).

### Detection of double cropping

Our field campaigns were conducted during mid to late summer, which allowed us to observe early-year cultivation patterns. However, in some locations, crop rotation is a common practice and single observations can misinform land cover mapping applications. To address this, we visually inspected the temporal variability of each sample to identify and interpret second harvest events. To do so, we used all available collection 1 Landsat Data (i.e. Landsat 4, 5, 7 and 8) for each of the sampling years (+/−2 months). Then, for each sampling site, we estimated the Normalized Difference Vegetation Index (NDVI) and masked cloud and shadows using the pixel quality information provided with the data. Finally, we derived an equidistant time series linearly interpolated every 16 days with consideration for the acquisition date of each pixel. When interpolating missing values, we searched for pixels acquired within 60 days before and after the target date. This was meant to avoid the over generalization of the time series, preserving relevant phenological patterns.

**Fig. 3:**
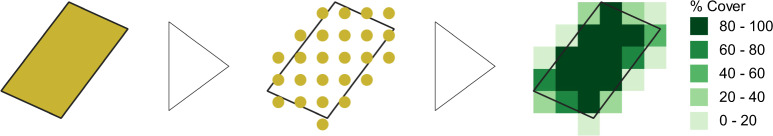
Spatial homogeneity of field samples. Translation of a field polygon (left) into centroid coordinates (centre), where each point reports on percent overlap between the respective pixel and the polygon (right). The Overlap is controlled by the pixel resolution. The higher the resolution, the higher the proportion of pixels with a high homogeneity.

Then, for each sample polygon, we extracted an average NDVI time-series for the corresponding sampling year. When computing the average, we considered the NDVI heterogeneity within each pixel. First, we used *poly2sample()* from the fieldRS R package (https://CRAN.R-project.org/package=fieldRS) to convert each polygon into points, where each point is a pixel that overlaps with the polygon. Additionally, the function reports on the percent overlap between a pixel and the polygon (Fig. 3). These data are used by *extractTS()* from the CAWaR package (https://cran.r-project.org/package=CAWaR), which derives an average for each time-step weighed by the percent cover of each pixel. This step accounts for mixed-pixel effects. At the edges of a field, a pixel may be composed by different crop types and contaminate the observed spectral signature when computing a simple average.

**Fig. 4:**
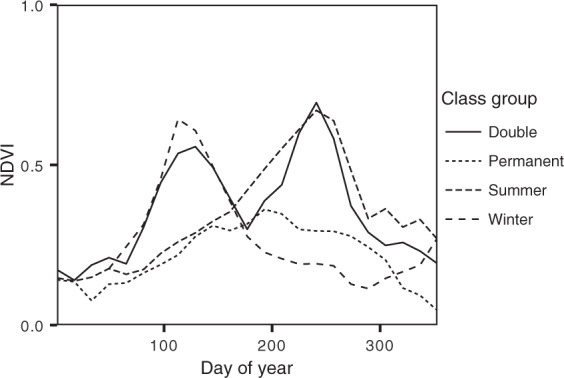
Comparison of phenological classes. Comparison of profiles for different phenological class groups. This information is used as a reference to re-classify crop types based on their respective NDVI profiles. “Winter” includes only winter wheat, which is planted in the last months of the year. “Summer” includes e.g. cotton and maize, which are planted at the begging of the year and usually harvested in mid- to late-summer. “Double” describes the existence of crop rotation (i.e. two phenological peaks) while “Permanent” depicts persistent agricultural practices, such as orchards.

After deriving the NDVI profiles for each polygon, we visually inspected them to detect double cropping events, expressed as two peaks in the NDVI time-series. When this phenomenon occurred, we interpreted the second peak with the support of local experts, as well as our own knowledge on the phenological behaviour of each crop. We supported our visual assessment with median profiles for each crop type derived with the *analyzeTS()* function from CAWaR, derived on a regional basis. Using the median, we exclude temporal outliers related to abnormal growth patterns and the mislabelling of samples. During our assessment, when the second phenological peak displayed a known pattern, we relabelled our samples accordingly. For example, if the first phenological peak (which we sampled on the field) corresponded to “wheat” and the second to “cotton”, we relabelled our sample as “wheat-cotton”. Following this process, we grouped our samples into phenological classes based on their NDVI curves (Fig. 4). When an interpretation was not possible due to data gaps or noise, we relabelled our sample as e.g. “wheat-other”. Samples to which we could not assign a phenological class objectively were labelled as “unclear”.

## Data Records

Our data are provided through figshare^28^ and is and contains three main files*: CAWa_cropTypes_samples.shp* (Table 1) containing the file samples; *CAWa_cropType_polygon-info.csv* (Table 2) containing quality information on each sample; *CAWa_cropType_time-series.csv* (Table 3) containing the sample-specific NDVI time-series used in the detection of double cropping events. The entries in *CAWa_PolygonInfo.csv* and *CAWa_cropType_time-series.csv* have the same sorting as the entries in *CAWa_cropTypes.shp* making them interoperable. To ease the combination of these data, we provide an RDS file named *CAWa_cropType.rds*, which compiles all resources through a list object usable in R (Table 4).

**Table 1:** Attributes of “CAWa_cropType_samples.shp”.

Column	Format	Description
sampler	Character	Institution leading the field campaign
country	Character	Country of sampling
region	character	Geographic region of sampling
date	numeric	Sampling date (format: yyyy-mm-dd)
year	character	Sampling year
label_1	character	Crop type; double cropping is labeled as CROP1-CROP2 (e.g. “wheat-other”)
label_2	numeric	Phenological class
area	character	Polygon area in m^2^

Description of the ESRI shapefile containing polygons of field samples on crop types.

**Table 2:** Attributes of “CAWa_cropType_polygon-Info.csv”.

Column	Format	Description
min.cover	Numeric	Minimum percent overlap between the polygon overlap between the polygon and the underlying 30 × 30 m Landsat pixels
max.cover	Numeric	Maximum percent overlap between the polygon and the underlying 30 × 30 m Landsat pixels
mean.cover	Numeric	Mean percent overlap between the polygon and the underlying 30 × 30 m Landsat pixels
count	Numeric	Number of 30 × 30 m pixels in a polygon

These data inform on the pixel homogeneity of each polygon in the sample dataset, based on 30 × 30 m satellite Data.

**Table 3:** Content of “CAWa_cropType_time-series.csv”.

Column	Format	Description
1, …, 353	Numeric	NDVI for a given day of year (given by the column name)

These data contain the weighted-mean NDVI time-series used in the validation of the crop-type labels initially assigned to each sample during the respective field surveys. For each row, corresponding to a unique sample, the 23 columns provide equidistant NDVI values with a 16-day interval. The name of each column informs on the day of the year in which the respective sample was collected.

**Table 4:** Content of “CAWa_CropType_samples.rds”.

List element name	R Class	Description
samples	SpatialPolygonsDataFrame	Content described in Table 1
Info	Data.Frame	Content described in Table 2
ndvi	Data.Frame	Content described in Table 3

The data described in Tables 1–3 are combined into an RDS file, which is an R specific file format. When read into R, the input data are organized in a list composed by three elements as described in the present table.

## Technical Validation

To guaranty the consistency of our ground-truth data, we first checked class labels for misspellings using *labelCheck()* from *fieldRS*. The function provided us with a list of unique classes within a pre-defined table structure meant for manual editing. We visually assesse each unique label looking for misspelling and needed class aggregations (e.g. “fruit trees” and “apple trees” merged as “orchard”). Then, we provided the table with corrected labels to *labelCheck()* to automatically correct the originals. We repeated this process after the detection of double cropping events to find labelling errors introduced during the visual assessment of temporal profiles. Additionally, during the visual assessment, we clearly mislabelled samples. These relate e.g. to cotton samples labelled as wheat that had visible summer peak in the NDVI. Here, we used our knowledge on crop-specific phenology behaviour, and the reference plots derived by *analyzeTS()* to consolidated our expectation on the shape of the temporal NDVI profile for a given class.

## Usage Notes

We developed this dataset to map the distribution of crop types in irrigation systems of the Aral Sea Basin in Central Asia. However, the use of these data can be extended. In the scope of land cover mapping, our data can support existing mapping services such as the Climate Change Initiative (CCI) land cover data (https://www.esa-landcover-cci.org/) as it complements spatial and temporal data gap in a large agricultural system. Since our samples are provided as polygons, users can freely adjust them to different satellite sensors. When dealing with high-resolution sensors, our pixel-based sampling approach can be used to translate our database into larger number of samples with associated information on pixel homogeneity. This information is an essential element in evaluating the consistency of land cover mapping applications within and between classes. On the other hand, when using mid and low-resolution satellite data, the sample homogeneity data provided with our product can be used as a metric of uncertainty.

Practitioners interested in collecting new samples might also profit from our data. In particular, users can rely on the NDVI time-series associated to each sample – as well as the samples themselves – as a reference when interpreting remotely-sensed data.

## Data Availability

The code used in the processing of our ground-truth data is open-source and was published in the Comprehensive R Archive Network (CRAN). The workflow for the processing of ground-truth data are available online in the form of an html vignette (https://CRAN.R-project.org/package=CAWaR/vignettes/CAWaR.html) and can be tested using example ground-truth data provided within the corresponding R package.
